# Adverse health outcomes and patient and physician perspectives of attenuated androgen use in hereditary angioedema^☆^

**DOI:** 10.1016/j.waojou.2026.101471

**Published:** 2026-09-22

**Authors:** Markus Magerl, Anete S. Grumach, Noemi Fluetsch Brennan, Adam Gordois, Maria Pedrosa, Nikolina Boskovic, Lisa Bloudek, Sandra Nestler-Parr, Patrick Gillard, Marcin Stobiecki, Sorena Kiani-Alikhan

**Affiliations:** a Institute of Allergology, Charité–Universitätsmedizin Berlin, Corporate Member of Freie Universität Berlin and Humboldt-Universität zu Berlin, Berlin, Germany; b Fraunhofer Institute for Translational Medicine and Pharmacology ITMP, Immunology and Allergology, Berlin, Germany; c Centro Universitario Faculdade de Medicina ABC, Santo André, SP, Brazil; d Curta Inc, Seattle, WA, USA; e BioCryst, Durham, NC, USA; f Department of Clinical and Environmental Allergology, Jagiellonian University Medical College, Krakow, Poland; g Royal Free London NHS Foundation Trust, London, UK

**Keywords:** Adverse effects, Androgens, Angioedemas, Hereditary, Attitude to health, Prevention and control

## Abstract

Attenuated androgens (AAs) historically have been used as long-term prophylaxis (LTP) for hereditary angioedema (HAE), and they continue to be used, especially in regions where access to newer therapies remains restricted. This systematic literature review assessed the adverse health outcomes associated with AAs, including adverse events (AEs), comorbidities, cardiovascular (CV) risk, as well as patient and physician perspectives. In total, 99 unique studies were identified through MEDLINE and EMBASE (searches up to March 2025), of which the majority were conducted in Europe (n = 57) and the United States (n = 26). Commonly reported adverse outcomes included weight gain, menstrual irregularities, virilization, myalgia, acne, and liver damage. Long-term use (≥5 years) of AAs, even at low doses, was associated with coagulation activation, hypertension, hypercholesterolemia, and hepatocellular carcinoma, a rare but serious AE. Studies also noted unfavorable changes in lipid profiles and markers of inflammation and endothelial dysfunction. Patients and physicians highlighted concerns over tolerability, AEs, and poor adherence. Notably, based on 3 serial surveys, physician reluctance to prescribe AAs rose from 18% in 2010 to 60% in 2019, likely reflecting the availability of alternative options. These findings underscore the need for improved access to targeted therapies for HAE and more research into the long-term CV risks of AAs.

## Introduction

Hereditary angioedema (HAE) is a rare genetic disorder marked by recurrent episodes of swelling in various parts of the body, including the hands, feet, face, and airway.^1^^,^^2^ Swelling of the larynx can be life-threatening. HAE is categorized into 3 types depending on the associated deficiency or dysfunction of the C1 inhibitor (C1INH) in circulation.^3^, ^4^, ^5^, ^6^ HAE type 1 is marked by deficient levels of circulating C1INH protein, whereas HAE type 2 is associated with normal C1INH protein levels exhibiting decreased C1INH functional activity due to dysfunctional C1INH protein. HAE patients with normal levels of functional C1INH (HAE-nC1INH) have also been described in the literature.^4^^,^^6^, ^7^, ^8^ HAE is estimated to affect approximately 1 in 50,000–100,000 people.^9^, ^10^, ^11^

The management of HAE includes on-demand treatment and short- and long-term prophylactic treatment (LTP), with the goal of normalizing patients’ lives.^10^^,^^12^ Prior to the introduction of targeted LTP in the late 2010s, attenuated androgens (AAs) were 1 of the few options commonly available for HAE prophylaxis.^13^ A placebo-controlled trial conducted in the 1970s demonstrated a reduction in attack frequency among danazol-treated patients.^14^ However, since the emergence of novel targeted LTPs, the standard of care has shifted away from AAs in favor of more effective and better tolerated treatment options.^15^ The 2021 World Allergy Organization/European Academy of Allergy and Clinical Immunology (WAO/EAACI) guidelines recommend plasma-derived C1-inhibitors, lanadelumab, and berotralstat as first-line treatments for HAE LTP.^10^ Attenuated androgens are considered second-line, meaning they are used only when first-line treatments are unavailable.^10^ In line with current treatment recommendations, AAs are now used less frequently; however, familiarity with AAs and limited access to newer, targeted prophylactic treatments, due to both regulatory and insurance-related barriers, have resulted in continued use of AAs in some patients.^16^^,^^17^

As synthetic derivatives of testosterone, AAs are associated with safety concerns, particularly among children and adolescents, and female patients with HAE.^10^ In younger patients, AAs may negatively impact bone development and potentially trigger precocious puberty.^10^^,^^18^ Women are disproportionally affected by adverse health consequences associated with the use of AAs for HAE LTP due to their effects on sex hormones.^10^^,^^19^^,^^20^ The use of exogenous androgens, such as AAs, for HAE LTP may lead to elevated androgen levels and consequent virilization in females. Virilization can encompass multiple effects, such as menstrual disturbances, altered libido, voice changes, increased body hair growth, clitoral hypertrophy, increased muscle mass, or reduction of breast size.^20^

In a systematic literature review (SLR) of long-term outcomes associated with AA use in patients with HAE, female patients regularly reported experiencing virilization, including menstrual irregularities, acne, and hirsutism.^4^ Riedl et al further found that while AAs provided prophylactic efficacy, adverse events (AEs) were common.^4^ In addition to commonly reported adverse outcomes such as weight gain, menstrual irregularities, and virilization, increased risks of hypertension, hypercholesterolemia, cardiovascular (CV) disease, and hepatocellular carcinoma have been reported with prolonged exposure to AAs.^4^^,^^21^ The effects of AA withdrawal also have the potential to result in complications and side effects.^22^, ^23^, ^24^, ^25^, ^26^

The objective of this study was to provide a comprehensive and contemporary evaluation of the adverse health outcomes of AA prophylaxis in patients with HAE, as well as patient and physician attitudes and perceptions of risk regarding AA use, to inform clinical decision-making and optimize patient care.

## Methods

### Data sources and search strategy

An SLR was conducted to identify all studies that provided information on adverse consequences of AA prophylaxis in HAE or patient and physician perspectives on use of AAs in HAE. The study was performed in accordance with the Preferred Reporting Items for Systematic Reviews and Meta-Analyses (PRISMA) for evidence synthesis and decision-making.^27^ MEDLINE (via PubMed) and EMBASE were searched for articles published in any language since database inception to March 24, 2025. The reference lists for previously conducted reviews that were identified via the database searches and reported quantitative data on adverse health outcomes were also reviewed for additional relevant studies. Please refer to Supplemental Table A and B for details on the search strategy and Supplemental Table C for the PRISMA checklist.

### Study selection and data extraction

Prospective studies, retrospective studies, and case reports/series were included if they reported AEs, comorbidities, changes in CV risk factors (such as lipid changes associated with AA treatment), or CV events in HAE patients on prophylaxis with AAs (danazol, stanozolol, oxandrolone, and methyltestosterone). Studies of patient and physician attitudes and perception of risk regarding AA prophylaxis were also included. Studies that investigated androgen use in sports and athletics were excluded, as were studies providing only a qualitative description of AEs without quantitative values and/or a data reference. Because of differences in baseline AA dose, study design, timepoint of assessment, and variation in AA treatment patterns across the globe, results were described qualitatively.

Publications identified from the search were dual-screened, using a two-stage process: title and abstract and full-text screening. Data extractions were completed by 2 independent investigators. Discrepancies or conflicts were resolved by consultation with a third independent investigator as needed.

## Results

### Study characteristics

After removal of duplicates, 1118 publications underwent title/abstract screening, and 529 were retained for full text screening. A total of 124 publications were deemed eligible for inclusion, including 27 previously conducted literature reviews that were not included in this SLR, but were retained to manually screen for additional references. Five additional records were included by hand searching the citations of previously published reviews, for a total of 129 publications (Fig. 1). Overall, 102 publications pertaining to 99 studies, including seven clinical trials, 52 observational studies, and 40 case reports/series were included in this SLR.

**Fig. 1:**
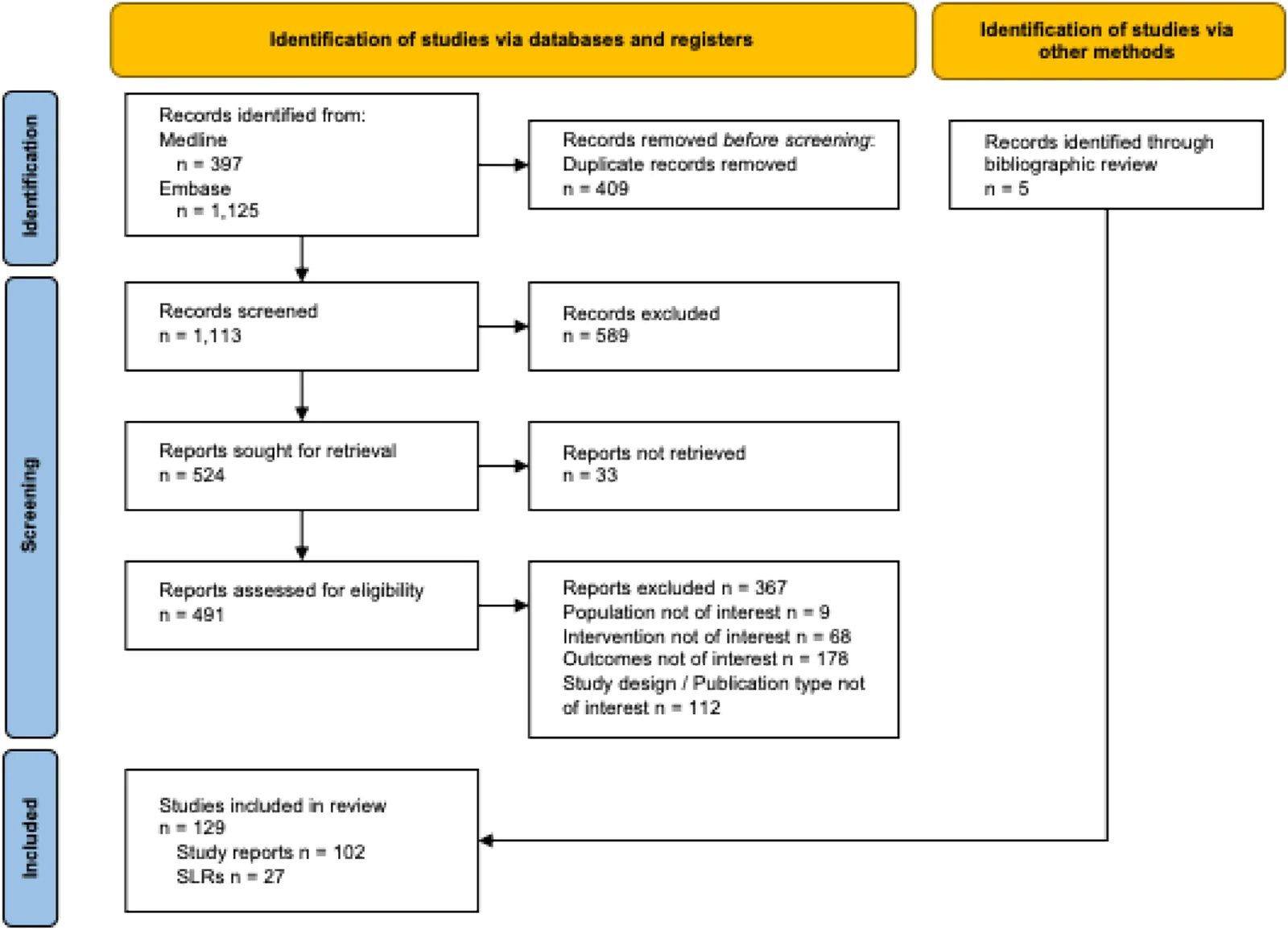
**PRISMA**. Note: Systematic literature reviews were not included in the final study count and were only utilized to manually screen for additional references.

Among the included publications, several were related but considered different studies. For example, Sloane et al (2007) conducted a follow-up study to the earlier study by Sheffer et al (1987).^28^^,^^29^ In another instance, Varga et al (2007) and Szegedi et al (2008) included a subset of the population originally examined by Széplaki et al (2005).^30^^,^^31^^,^^32^ Similarly, Liu et al (2019) and Liu et al (2020) recruited participants from the same patient pool.^33^^,^^34^ Barakat (1999) provided additional data and extended follow-up to the same case first described in Barakat 1993.^35^^,^^36^ Likewise, Bork et al (2002) reported on additional outcomes and extended follow-up on patients described in Bork et al (1999), while also reporting on an additional case.^37^^,^^38^ Finally, the cases described in Hintner et al (1979) included 1 previously described in Tappeiner et al (1979).^39^^,^^40^

Of the 99 included studies, 76 studies reported on treatment patterns, 76 studies reported on adverse health outcomes, 13 studies reported on CV risk factors and events associated with AA use, and 28 studies reported on patient and physician perspectives. Study characteristics of included clinical trials and observational studies are provided in Table 1 and Table 2. Study characteristics of included case reports/series are provided in Supplemental Table D.

**Table 1:** Included clinical trials.

Citation	Study design	Geography	Follow-up duration	N^a^	Intervention^b^	Age, years	Female	Duration of AA use, years
						Mean (SD) or median [range]	%	Mean [range]
Birjmohun 2008^41^	Short-term crossover trial	The Netherlands	12 weeks	15	Danazol/Placebo (healthy volunteers)	32.0 [IQR: 27.3, 33.0]	0	– [4, 8] weeks
	Long-term cross-sectional trial	The Netherlands	–	17	Danazol	37.0 [IQR: 33.5, 46.0]	58.8	13.9 [5.0, 30.0]
				17	No danazol (healthy controls)	37.5 [IQR: 34.3, 46.5]	58.8	–
Gelfand 1976^14^	Crossover trial	United States	6–11 months	9	Danazol/Placebo	33 [25, 63]	55.6	–
Hosea 1980^42^	Prospective open-label	United States	1–6 years	69	Danazol	–	–	–
Rothbach 1979^43^	Clinical trial	United States	Mean: 15 months	4	Danazol	– [27,46]	75.0	–
Sheffer 1979^44^	Dose-finding study	United States	May 1976–October 1977	16	Oxymetholone	–	50.0	–
Sheffer 1977^45^	Crossover trial	United States	Mean: 14.5 months	4	Methyltestosterone/Placebo	–	0	–
Sweet 1980^46^	Clinical trial	United States	Mean: 22.1 months	11	Danazol	– [16, 71]	54.5	–

Dashes (−) indicate data not reported/not applicable.

Key: AA, attenuated androgen; HAE, hereditary angioedema; IQR, interquartile range; N, total number; RCT, randomized clinical trial; SD, standard deviation; US, United States of America.

a HAE patients and/or healthy controls receiving treatments other than AA are not included.

b HAE patients unless otherwise indicated

**Table 2:** Included observational studies. Dashes (-) indicate data not reported/not applicable.

Citation	Study design	Geography	Follow-up duration	N^a^	Intervention^b^	Age, years	Female	Duration of AA use, years
						Mean (SD) or median [range]	%	Mean (SD) or [range]
Agostoni 1992^47^	Retrospective observational	Italy	1973–January 1992	59	Danazol or stanozolol	–	–	4.8 (3.4)
Agostoni 1980^48^	Prospective observational	Italy	≥25 months	14	Danazol: 57.1% Stanozolol: 14.3% Danazol & stanozolol: 28.6%	– [25, 58]	57.1	– [0.5, 2]
Baptist 2024^49^	Cross-sectional (patient survey)	United States	–	17	Any	65.2 (3.7)	82	–
Bork 2008^50^	Retrospective observational	Germany, Denmark	–	118	Danazol	–	–	11.0 (7.9)
Bouillet 2022^51^	Cross-sectional (physician survey)	France	–	120	AAs	–	–	–
Cao 2021^52^	Cross-sectional (patient survey)	China	–	77	Danazol	At start of danazol treatment: 33.3 (13.6) At end of study: 50.0 (14.7)	49.2	–
Cicardi 1997^53^	Retrospective observational	Italy	–	36	Danazol & stanozolol: 86.1%Danazol: 13.9% Stanozolol: 0%	44 [22, 74]	77.8	Median [range]: 10.5 [1.8, 22.8] years
				34	No AAs	– [15, 67]	54.5	–
Cicardi 1991^54^	Prospective observational	Italy	≤13 years	61^c^	Stanozolol: 50.8%Danazol: 32.8%Stanozolol and danazol: 16.4%	–	67.2	– [<1, ≥10]
Cicardi 1983^55^	Prospective observational	Italy	15–47 months	13	Stanozolol: 84.6%Danazol: 15.4%	– [28, 59]	69.2	Stanozolol: – [4, 30] months Danazol: – [15, 30] months
Coovadia 2018^56^	Retrospective observational	South Africa	–	21	Danazol	–	76.2	–
Demir 2019^57^	Retrospective observational	Turkey	Mean: 63.51 months	43	Danazol	–	–	–
Demirtürk 2012^58^	Cross-sectional case-control	Turkey	January–July 2010	26	Danazol	37.6 (13)	53.8	41 (35) months
				30	No danazol (healthy controls)	38.4 (9)	50.0	–
Dispenza 2012^59^	Cross-sectional (physician survey)	Global	–	Physicians (n = 201)	Most frequently prescribed prophylaxis (n = 191) STP:High-dose AAs: 19% LTP: Danazol: 64%Stanozolol: 9% Oxandrolone: 1%	–	–	–
Ebo 2023^60^	Cross-sectional (physician + patient survey)	Belgium	–	Expert panel (NR) Patients (n = 125)	LTP (including AAs, TXA, pdC1INH concentrate)	–	–	–
Farkas 2016^61^	Retrospective observational	Hungary	29 years	65	Danazol	–	–	–
Farkas 2012^62^	Prospective observational	Hungary	0.6–30 years	38	Danazol (STP)	–	–	–
Farkas 2010^63^	Retrospective observational	Hungary	5 years	46	Danazol	32.5 [9, 62]	52.2	≥4
				46	No danazol	16 [2, 73]	67.4	–
Farkas 2002^64^	Retrospective observational	Hungary	1–19 years	8	Danazol	–	–	– [2, 11]
Fu 2018^65^	Cross-sectional (physician survey)	Canada	–	Physicians (n = 34)	LTP danazol: 41.9% ODT danazol: 5.9%	–	–	–
Jindal 2021^66^	Retrospective observational	India	Mean: 57 months	N = 20 AAs before/during puberty (n = 15) AAs after puberty (n = 5)	Stanozolol alone: 20%Stanozolol and TXA: 80%	– [8, 60]	35.0	–
Jose 2018^67^	Cross-sectional (patient survey)	United States	–	22	AAs	–	–	–
Kalaria 2013^68^	Cross-sectional (physician survey)	Global	–	Physicians (n = 66)	AAs	–	–	–
Kőhalmi 2016^18^	Prospective case-control	Hungary	5 years	39	Danazol	– [18.00, 66.46]	53.8	1
				33	Danazol	– [18.00, 47.29]	51.5	3
				30	Danazol	– [18.00, 47.29]	50.0	5
Kreuz 2009^69^	Retrospective observational	Germany	–	22	Danazol	Female: 41.8 (10.1)Male: 35.8 (7.3)	59.1	11.25 [0.5, 23.5]
Lindsay 2022^70^	Retrospective observational	New Zealand	12 months	18	Danazol/Stanozolol	–	–	–
Liu 202,0^33^^d^	Cross-sectional (patient survey)	China	–	89	Danazol	–	–	–
Liu 201,9^32^^d^	Cross-sectional (patient survey)	China	–	74	Danazol	–	–	–
Lumry 2024^71^	Cross-sectional (physician survey)	United States	–	Physicians (n = 5)	AAs	–	–	–
Lumry 2010^72^	Cross-sectional (patient survey)	United States	–	226	AAs	–	70.8	–
Magerl 2023^73^	Cross-sectional (patient survey)	Germany	–	99	Any	–	–	–
Mendivil 2023^74^	Retrospective observational	United States, Canada, Germany, Australia, United Kingdom, Italy	Mean: 5.5 years	75	LTP:Danazol: 86.7% Stanozolol: 9.3%Oxandrolone: 4.0% STP:Danazol: 14/54 (25.9%) ODT:Danazol: (n = 7)	–	–	–
Nordenfelt 2016^75^	Cross-sectional (patient survey)	Sweden	–	38	AAs	–	–	–
Pedrosa 2009^76^	Retrospective observational	Spain	–	33	AAs (mostly stanozolol)	–	–	–
Psarros 2014^77^	Retrospective observational	Greece	3 years	67	Danazol	–	–	– [6 months, 32 years]
Riedl 2011;^78^	Cross-sectional (physician survey) – 2010	United States	–	Physicians (n = 172)	LTP:Danazol: 55.8% Stanozolol: 10.5% Oxandrolone: 2.9% STP: Androgens: 18.6%	– [<15, 75]	–	–
Riedl 2015;^79^	Cross-sectional (physician survey) – 2013	United States	–	Physicians (n = 245)	LTP: Danazol: 23%Stanozolol: 4%Oxandrole: 2% STP: Androgens: 8.9%	–	–	–
Riedl 2021a^80^	Cross-sectional (physician survey) – 2019	United States	–	Physicians (n = 156)	LTP: Danazol: 6% Stanozolol: 1% Oxandrole: 0% STP: Androgens:	–	–	–
Riedl 2021b^81^	Cross-sectional (physician survey)	United States	–	Physicians (n = 109)	–	–	–	–
	Cross-sectional (patient survey)	United States	–	75	HAE prophylaxis	44.1 (13.4)	80	15.1 (12.0)^h^
Sheffer 198,7^28^^e^	Retrospective observational	United States	10 years	37	Stanozolol	– [9, 73]	62.2	–
Sloane 200,7^29^^e^	Retrospective observational	United States	≥20 years	22	Stanozolol	Mean [range]: 60.3 [31, 88]	59.1	– [23, 29]
Steiner 2016^82^	Questionnaire survey within a cohort study	Switzerland	1 year	26	Danazol (total)	Mean [range]: 44 [5, 80]	50	–
				78	No danazol (total)	Mean [range]: 44 [5, 80]	56.4	–
				13	Danazol (men)	–	0	–
				34	No danazol (men)	–	0	–
				13	Danazol (women)	–	100	–
				44	No danazol (women)	–	100	–
Szegedi 2008^32^	Cross-sectional case control	Hungary	20–288 months	32	Danazol	37.2 [IQR: 25.61, 45.51]	50	–
				25	No danazol	31.07 [IQR: 19.64, 43.42]	76	–
				20	No danazol (healthy controls)	38.67 [IQR: 25.92, 55.89]	60	–
Széplaki 200,5^30^^f^	Retrospective observational	Hungary	December 2000–November 2003	37	Danazol	40.0 [IQR: 32.0, 47.5]	51.4	Median [IQR]: 45.1 [23.4, 84.9] months
				27	No danazol	36.0 [IQR: 22.0, 49.0]	66.7	Median [IQR]: 25.08 [19.6, 44.3] months
				66	No danazol (healthy controls)	36.0 [20.0, 49.0]	57.6	–
Tse 2017^83^	Retrospective observational	United States	Mean: 18.4 years	36	AA-exposed	30.4 (18.5)	58.3	–
			Mean: 17.6 years	180	Matched controls for AA-exposed^g^	30.7 (18.2)	58.3	–
			Mean: 14.1 years	14	Non-AA-exposed	11.1 (14.71)	64.3	–
			Mean: 14.0 years	70	Matched controls for non-aa-exposed^g^	11.3 (14.47)	64.3	–
Varga 200,7^31^^f^	Retrospective observational	Hungary	–	33	Danazol	39.6 [IQR: 32.2, 47.7]	54.5	– [5, 195] months
				26	No danazol	35.1 [IQR: 20.4, 46.7]	69.2	–
Winnewisser 1997^84^	Retrospective observational	Switzerland	–	11	Danazol: 81.8% Stanozolol: 18.2%	–	–	–
Xu 2022^85^	Retrospective observational	China	–	74	Danazol	– [16, 71]	52.7	– [2, 26]
Yong 2024^86^	Cross-sectional (physician + patient survey)	United Kingdom	–	Healthcare professionals (n = 59) Patients (n = 39)	AAs	–	–	–
Zanichelli 2024^87^	Retrospective observational	Italy	≥43 years	218	Prophylaxis (danazol, stanozolol, TXA)	56.5 (17.2)	58.3	–
				228	No prophylaxis	48.7 (18.8)	46.1	–
Zotter 2014^20^	Cross-sectional (patient survey)	Hungary	–	31	Danazol	49 [20, 70]	100	10.31 [2, 23]
			–	41	No danazol (healthy controls)	48 [32, 63]	100	–
Zuraw 2016^88^	Cross-sectional (patient survey)	United States	–	180	Prior AA use	41.5 (SEM: 0.9)	77.8	–
			–	344	Current AA use	45.1 (SEM: 0.7)	64	–
			–	524	Prior or current use of AAs	–	68.7	–
			–	126	Never used AAs	37.4 (1.1)	81	–
Zurlo 1990^89^	Retrospective observational	United States	≥6 months	60	Danazol	Mean [range]: 35.2 [17, 74]	100	Mean (SEM): 59.7 (4.5) months

Key: AA, attenuated androgen; HAE, hereditary angioedema; IQR, interquartile range; LTP, long-term prophylaxis; n, number; N, total number; NR, not reported; ODT, on-demand treatment; SD, standard deviation; SEM, standard error of the mean; STP, short-term prophylaxis; TXA, tranexamic acid; US, United States of America.

a HAE patients and/or healthy controls receiving treatments other than AA are not included.

b HAE patients unless otherwise indicated.

c Five patients were not available for required follow-up evaluations and were excluded.

d Liu 2019 and Liu 2020 recruited from the same patient pool.

e Sloane 2007 is a follow-up study to Sheffer 1987.

f Varga 2007 is a subgroup study of the population included in Széplaki 2005.

g Matched control groups (healthy controls) did not receive AA treatment

h Any therapy.

The vast majority of included studies focused on adults with HAE or included both adults and children without stratifying the results. Only 1 prospective observational study in Hungary included both adults and children, with mean ages of 41.3 (range: 18.5–81.2) years and 12.2 (range: 2.2–17.3) years, respectively.^62^ The proportion of female patients with HAE varied widely across studies (0%–100%); 1 cross-over trial evaluated only males.^45^ and 3 observational studies specifically assessed AEs in female HAE patients, or provided stratified results.^20^^,^^82^^,^^89^ Geographically, the studies were distributed across various regions. Most studies were conducted in Europe, followed by the United States (US) and Asia (Fig. 2).

**Fig. 2:**
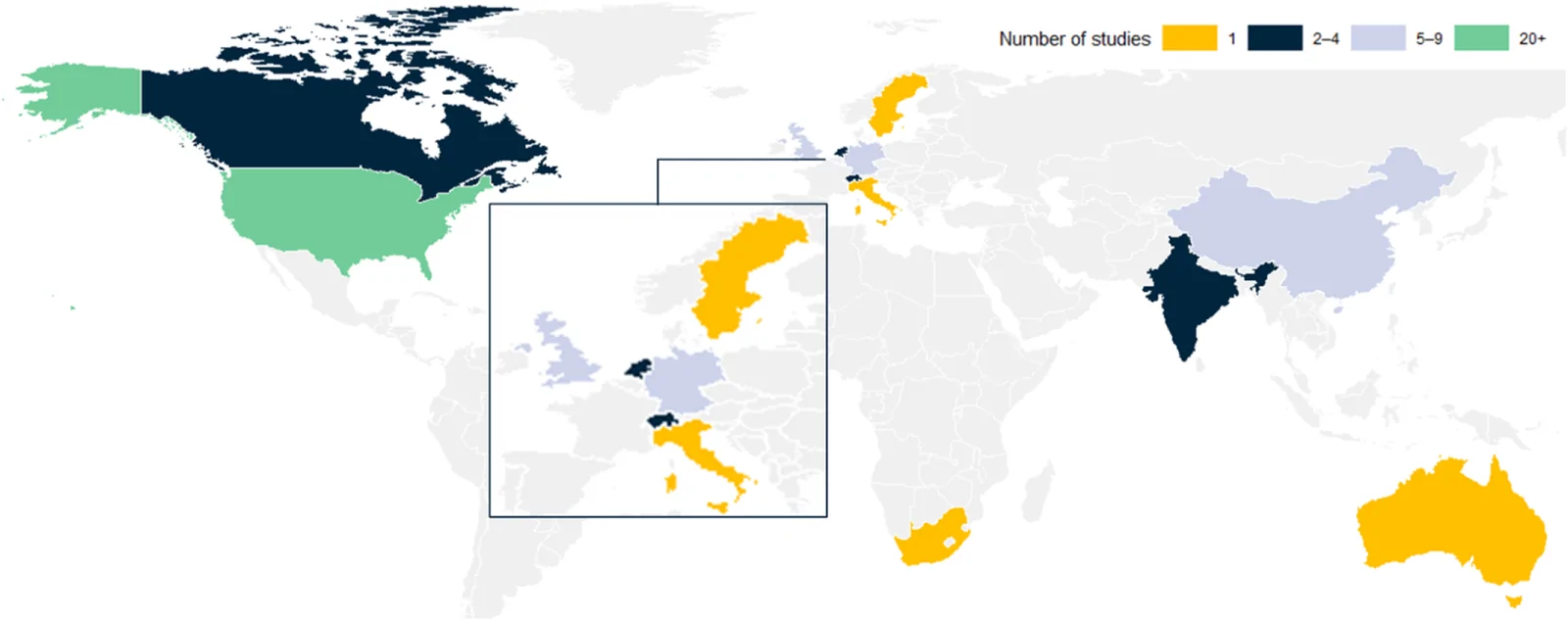
**Study distribution by country**. Note: Global studies that do not provide country-specific data, or studies where geography is not reported, are not included in this figure.

The duration of LTP for HAE with AAs spanned from approximately 6 months to more than 30 years. Danazol was identified as the most frequently used AA and was the treatment of interest in 6 clinical trials, 21 observational studies, and 34 case reports/series. Stanozolol was evaluated in 5 observational studies and 1 case report/series, while oxymetholone and methyltestosterone were evaluated in 1 clinical trial each. The remaining studies (25 observational studies and 6 case reports/series) reported either on mixed treatment regimens or did not specify the AA used. The median daily dose of AAs varied across studies, ranging from 50 mg to 600 mg for danazol and 0.5 mg–6 mg for stanozolol. Often, treatment was initiated at a higher dose, which was tapered down to the minimum effective dose.^20^^,^^43^^,^^46^^,^^48^^,^^54^^,^^74^^,^^85^^,^^87^^,^^89^ The use of danazol and stanozolol for HAE short-term prophylaxis before dental procedures was highlighted in several retrospective case reports/series.

### Adverse effects of attenuated androgen use

Among the most frequently reported AEs associated with AA use were increased body weight, menstrual irregularities, liver damage, acne, psychological morbidity, CV morbidity, hirsutism, and hypertension (Table 3, Table 4, Table 5, Table 6, Table 7). In particular, 1 retrospective study conducted in Italy with a follow-up duration of up to 43 years found that LTP with AAs (compared to no LTP) appeared to be a risk factor for various AEs and morbidities, including hypertension (22.8% vs. 10.8%; odds ratio [OR] 2.02), hypercholesterolemia (19.5% vs. 5.3%; OR 3.97), diabetes mellitus (5% vs. 1.4%; OR 3.21), hepatic angioma (4.4% vs. 0.7%; OR 8.35), and focal nodular hyperplasia (2.5% vs. 0.4%; OR 6.9).^87^

**Table 3:** Reported adverse events with attenuated androgens in clinical trials & observational studies.

Adverse events [total studies, n]	Danazol [16]		Stanozolol [3]	Danazol and/or stanozolol [6]^a^	Oxymetholone [1]	AAs [8]^b^
	% patients, range [studies, n]		% patients, range [studies, n]	% patients, range [studies, n]	% patients, range [studies, n]	% patients, range [studies, n]
**Menstrual irregularities [23]**	50–100% [4]	23–79% [7]	9–57% [3]	38–80% [4]	38% [1]	36–52% [4]
**Increased body weight [22]**	38–100% [2]	19–62% [7]	14–41% [3]	24–39% [3]	–	5–71% [6]
**Acne [20]**	7–17% [3]	8–19% [5]	15–25% [2]	11–27% [3]	13% [1]	3–43% [6]
**Liver damage [17]**	0% [1]	3–48% [6]	10% [1]	0–39% [5]	–	0–29% [4]
**Psychological morbidity [16]**	0–16% [2]	1–77% [5]	10% [2]	5–27% [2]	13% [1]	26–60% [4]
**Headache [11]**	7–22% [2]	5–68% [6]	–	–	–	3–41% [3]
**Hypertension [11]**		2–23% [3]	5% [1]	23–25% [2]	–	3–38% [4]
**Hirsutism [10]**	8% [1]	12–42% [3]	4–57% [3]	27% [1]	–	3–49% [2]
**Myalgia/Muscle cramps [10]**	0–28% [2]	1–40% [5]	–	–	–	5–32% [3]
**Transaminase elevations [8]**	14% [1]	40–77% [2]	5% [1]	23% [1]	–	3–41% [3]
**Virilization [6]**^c^	0% [1]	1–32% [2]	–	0% [1]	–	0–22% [2]
**Hepatocellular carcinoma [2]**	–	–	–	0% [1]	–	0.1% [1]

Dashes (−) indicate data not reported/not applicable.

Key: AAs, attenuated androgens; n, number.

Note 1: clinical trials are highlighted in grey (n = 6).

Note 2: Related publications contributing to these study counts were treated as distinct studies; patient overlap therefore cannot be ruled out.327.

a Population includes mixed treatment.

b Includes studies that included all androgens and did not present stratified results.

c Only includes AEs reported as virilization (ie, does not include subcategories of virilization such as voice deepening)

**Table 4:** Reported adverse events with attenuated androgen in case reports/series.

Adverse events [total case reports/series, n]	Danazol [32]^a^	Danazol and/or stanozolol [3]^b^	Danazol and/or oxandrolone [2]	Stanozolol and/or TXA [1]^b^	Stanozolol and/or oxandrolone [1]^b^
	Number of patients/total patients [studies, n]	Number of patients/total patients [studies, n]	Number of patients/total patients [studies, n]	Number of patients/total patients [studies, n]	Number of patients/total patients [studies, n]
**Increased body weight [13]**	12/14 [9]	2/2 [2]	3/11 [2]	–	–
**Liver damage [10]**	9/27 [8]	–	1/11 [2]	–	–
**Menstrual irregularities [9]**	8/10 [8]	–	–	–	1/1 [1]
**Hirsutism [6]**	2/5 [3]	2/2 [1]^c^	–	1/1 [1]	1/1 [1]
**Virilization [6]**^d^	3/5 [4]	–	1/4 [2]	–	–
**Acne [5]**	4/6 [4]	–	–	1/1 [1]	–
**Hepatocellular carcinoma [4]**	4/4 [4]	–	–	–	–
**Hypertension [4]**	3/5 [3]	–	3/10 [1]	–	–
**Myalgia/Muscle cramps [4]**	5/5 [3]	–	2/10 [1]	–	–
**Psychological morbidity [4]**	5/5 [3]	–	–	1/1 [1]	–
**Transaminase elevations [4]**	3/3 [3]	–	1/10 [1]	–	–
**CV morbidity [3]**	1/1 [1]	1/1 [1]	2/10 [1]	–	–
**Headache [2]**	1/3 [1]	–	2/10 [1]	–	–

Dashes (−) indicate data not reported/not applicable.

Key: CV, cardiovascular; n, number; TXA, tranexamic acid.

Note: Related publications contributing to these study counts were treated as distinct studies; patient overlap therefore cannot be ruled out.

a One study (Gower 2012) included 2 patients treated with danazol and 1 patient for whom androgen type was unknown.

b Population includes mixed treatment.

c Daughters of patient (case 2) not included in analysis (Azzam 2015).

d Only includes AEs reported as virilization (ie, does not include subcategories of virilization such as voice deepening)

**Table 5:** Adverse events in female patients.

Citation	Female, n	Duration of AA use, range	Virilization, %	Individual virilization effects in clinical trials and observational studies (%) and case reports/series (n)					
				Menstrual irregularities	Hirsutism	Acne	Voice changes	Breast changes	Clitoral changes
**Clinical trials [n=5]**									
Gelfand 1976^14^	5	–	–	100	–	–	–	–	–
Hosea 1980^42^	50	–	–	50	–	8	10	–	–
Rothbach 1979^43^	3	13–17 months	–	100	–	–	–	–	–
Sheffer 1979^44^	8	–	–	37.5	–	12.5	25.0	–	–
Sweet 1980^46^	6	–	“Not significant”	100	–	16.7	–	–	–
**Observational studies [n=16]**									
Agostini 1980^48^	8	0.5–2 years	–	37.5	–	12.5	–	–	–
Bork 2008^50^	58	2 months - 30 years	67.2	29.3	–	–	29.3	10.3	10.3
Cao 2021^52^	44	–	–	75.0	13.6	15.9	11.4	18.2	–
Cicardi 1997^53^	22	1.8–22.8 years	–	68.2	–	–	–	–	–
Cicardi 1991^54^	41	<1 - ≥10 years	0	80^a^	–	–	–	2.4	–
Jindal 2021^66^	7^b^	4 months - 17 years	–	57.1	57.1	28.6	28.6	–	–
Kreuz 2009^69^	13	0.5–23.5 years	–	92.3	–	–	–	–	–
Liu 2020^34^	52	–	–	67.3	–	–	–	–	–
Lumry 2010^72^	160	–	–	51.9	–	–	–	–	–
Sheffer 1987^28^	23	–	–	8.7	4.3	–	4.3	–	–
Sloane 2007^29^	12^c^	23–29 years	–	25.0	–	25.0	8.3	–	–
Winnewisser 1997^84^	5	–	–	60.0	60.0	40.0	–	–	–
Xu 2022^85^	39	2–26 years	1.4	23.1	–	17.9	–	–	–
Zotter 2014^20^	31	2–23 years	84^d^	26	42	19	19	16	3
Zuraw 2016^88^	462	–	–	49	47	–	–	–	–
Zurlo 1990^89^	60	Mean: 5 years	–	79^e^	12	8	8	–	–
**Case reports/series [n=16]**									
Altman 2006^90^	1	6 weeks	–	–	–	1	–	–	–
Barakat 199,9^35^^f^	1	–	0	–	–	–	–	–	–
Barakat 199,3^34^^f^	1	–	0	–	–	–	–	–	–
Duhra 1990^91^	1	2 years	–	1	–	–	–	–	–
Farkas 2015^92^	1	25 years	–	1	–	–	–	–	–
Gower 2012^93^	3	1 year	–	–	1	1	1	–	–
Helsing 2006^94^	1	16 years	1	1	1	1	–	–	–
Hermann 2004^95^	1	2 years	–	1	–	1	–	–	–
Höpfl 1990^96^	1	10 years	–	0	0	–	1	–	–
Jindal 2024^97^	1	–	–	1	1	–	–	–	–
Lei 2011^98^	1	–	1	–	–	–	–	–	–
Marrawani 2024^99^	1	–	–	1	–	–	–	–	–
Maurer 2022^24^	3	14–29 years	1	–	–	–	–	–	–
Pastó-cardona 2004^100^	3	–	–	2	–	–	–	–	–
Suh 1995^101^	1	–	–	1	–	–	–	–	–
Weller 2016^102^	1	11 years	–	–	1	1	1	–	–

Dashes (−) indicate data not reported/not applicable.

Key: AA, attenuated androgens; n, number.

a 80% of pre-menopausal women (N is unclear).

b Study states 11 females but reports data for only 7.

c 13 females included, but 1 female did not complete the survey.

d Includes weight gain as a virilization effect.

e Denominator is among pre-menopausal women without a hysterectomy (n = 42).

f Barakat 1999 reports on the same case described in Barakat 1993 with additional follow-up.

**Table 6:** Cardiovascular risk factors.

Citation	Intervention^a^	Total cholesterol, mmol/L	HDL-C, mmol/L	LDL-C, mmol/L	Triglycerides, mmol/L	Creatinine, μmol/L	CRP, mg/L	hsCRP, mg/L
		Mean (SD) or median [IQR]	Mean (SD) or median [IQR]	Mean (SD) or median [IQR]	Mean (SD) or median [IQR]	Mean (SD) or median [IQR]	Mean (SD) or median [IQR]	Mean (SD)
**Clinical trials [n=1]**								
Birjmohun 2008^41^	Danazol (healthy volunteers)	–	1.0 (0.2)	2.6 (0.8)	0.7 (0.5)	–	–	1.7 (2.3)
	Placebo (healthy volunteers)	–	1.4 (0.3)	2.6 (1.0)	0.7 (0.5)	–	–	1.3 (1.2)
	Danazol	4.8 (1.0)	1.1 (0.5) pmol/L	3.3 (1.1)	0.9 (0.5)	–	–	1.5 (2.1)
	No danazol (healthy controls)	4.7 (0.9)	1.2 (0.5) pmol/L	3.1 (0.8)	1.1 (0.7)	–	–	1.6 (2.0)
**Observational studies [n=6]**								
Cicardi 1997^53^	Danazol/Danazol & stanozolol	213 [119, 302] mg/dL	–	–	–	Plasma creatinine: 0.83 [0.53–2.0] mg/dL	–	–
	No AAs	193.5 [141, 289] mg/dL	–	–	–	Plasma creatinine: 0.9 [0.6, 1.26] mg/dL	–	–
Demirtürk 2012^58^	Danazol	195 (44) mg/dL	25 (36) mg/dL	131 (38) mg/dL	118 (58) mg/dL	–	2.22 (1.33)	–
	No danazol (healthy controls)	183 (25) mg/dL	30 (44) mg/dL	119 (24) mg/dL	125 (34) mg/dL	–	1.79 (0.99)	–
Sloane 2007^29^	Stanozolol	Range: 109, 226 mg/dL	Range: 21, 59 mg/dL	Range: 42, 159 mg/dL	Range: 23, 182 mg/dL	–	–	–
Szegedi 2008^32^	Danazol	5.48 [4.69, 6.26]	0.90 [0.77, 1.24]	3.86 [2.74, 4.56]	1.03 [0.76, 1.68]	78.00 [73.50, 87.00]	1.56 [0.72, 5.40]	–
	No danazol	4.96 [4.21, 5.72]	1.45 [1.17, 1.75]	2.82 [2.01, 4.00]	0.88 [0.65, 1.48]	64.00 [54.50, 69.00]	1.11 [0.58, 4.79]	–
	No danazol (healthy controls)	4.73 [3.90, 5.31]	1.75 [1.41, 1.91]	2.49 [1.85, 2.88]	1.07 [0.76, 1.30]	65.00 [62.00, 74.75]	0.86 [0.52, 1.93]	–
Széplaki 2005^30^	Danazol	5.17 [4.24, 6.37]	1.10 [0.93, 1.37]	3.40 [2.81, 4.12]	1.20 [0.93, 1.54]	–	–	–
	No danazol	5.03 [3.97, 5.59]	1.38 [1.26, 1.74]	2.72 [2.04, 3.37]	0.89 [0.68, 1.80]	–	–	–
	No danazol (healthy controls)	5.11 [4.44, 5.81]	1.54 [1.29, 1.83]	2.79 [2.41, 3.64]	1.03 [0.76, 1.34]	–	–	–
Varga 2007^31^	Danazol	5.23 [4.37, 6.25]	1.08 [0.91, 1.38]	3.44 [2.93, 3.94]	1.22 [0.92, 1.55]	–	–	–
	No danazol	5.03 [4.04, 5.64]	1.38 [1.26, 1.75]	2.72 [2.06, 3.38]	0.89 [0.70, 1.66]	–	–	–

Dashes (−) indicate data not reported/not applicable. Key: AAs, attenuated androgens; CRP, c-reactive protein; HAE, hereditary angioedema; HDL-C, high-density lipoprotein – cholesterol; hs-CRP, high sensitivity C-reactive protein; IQR, interquartile range; LDL-C, low-density lipoprotein – cholesterol; n, number; SD, standard deviation.

a HAE patients unless otherwise indicated.

**Table 7:** Cardiovascular events.

Citation	Intervention^a^	Daily dose	Treatment duration	CV events, n (%)
**Observational studies [n=8]**				
Bork 2008^50^	Danazol	Mean [range]: 171.2 [40, 1000] mg	Mean (SD) [range]: 11.0 (7.9) years [2 months, 30 years]	Stroke: 1 (0.85) Myocardial infarction: 1 (0.85) Tachycardia: 1 (0.85)Thromboembolic disease: 1 (0.85)
Cicardi 1997^53^	Danazol/Danazol & stanozolol	1–2 mg/50–200 mg	Median [range]: 10.5 years [1.8, 22.8]	Myocardial infarction: 1 (3)
	No AAs	–	–	–
Lindsay 2022^70^	Danazol/Stanozolol	100–400 mg/2 mg	–	Ischemic heart disease: 1 (5.6)
Liu 2020^34^	Danazol	>200 mg: 34.6% 100–199 mg: 38.5% 0–99 mg: 26.9%	–	Heart symptoms: 2 (2.2)
Nordenfelt 2016^75^	AAs	–	–	No major CV events reported
Tse 2017^83^	AA-exposed	–	–	Cardiac disease (MI, CAD, or CHF): 1 (2.8) Stroke: 1 (2.8)
	Matched controls for AA-exposed^b^	–	–	Cardiac disease (MI, CAD, or CHF): 9 (5) Stroke: 11 (6.1)
	Non-AA-exposed	–	–	Cardiac disease (MI, CAD, or CHF): 0 (0) Stroke: 1 (7.1)
	Matched controls for non-aa-exposed^b^	–	–	Cardiac disease (MI, CAD, or CHF): 0 (0) Stroke: 1 (1.4)
Szegedi 2008^32^	Danazol	Median [IQR]: 100 [63.12, 148.78] mg	–	Manifestation of atherosclerosis: 1 (3.1)
	No danazol	–	–	Manifestation of atherosclerosis: 1 (4)
	No danazol (healthy controls)	–	–	Manifestation of atherosclerosis: 0 (0)
Zanichelli 2024^87^	Prophylaxis (danazol, stanozolol)	–	–	CV pathologies (no acute MI): 5 (2.5) Acute MI: 6 (3) Cerebrovascular pathologies: 8 (3.9)
	No prophylaxis	–	–	CV pathologies (no acute MI): 9 (2) Acute MI: 17 (3.8) Cerebrovascular pathologies: 11 (2.5)

Dashes (−) indicate data not reported/not applicable.

Key: AA, attenuated androgen; CAD, coronary artery disease; CHF, congestive heart failure; CV, cardiovascular; IQR, interquartile range; MI, myocardial infarction; n, number; SD, standard deviation; TXA, tranexamic acid.

a HAE patients unless otherwise indicated.

b Matched control groups (healthy controls) did not receive AA treatment.

Some adverse events commonly associated with AA can manifest within the first few months of treatment (eg, increased body weight, menstrual irregularities, and acne) while others appear to be more pronounced with longer-term exposure (eg, liver abnormalities).^42^^,^^83^^,^^88^ A retrospective cross-sectional study encompassing 650 patients with HAE found a significant positive correlation between AA dose and several AEs, most notably psychological morbidity, cramps, and menstrual abnormalities (p < 0.01).^88^ Similar relationships were observed between the duration of treatment and several AEs, such as elevated cholesterol and triglyceride levels and increased facial hair (p < 0.001). Liver function, hypertension, and headaches were significantly related to both dose and treatment duration (p < 0.05).

Similarly, in a study examining adverse health outcomes in US patients with HAE (n = 69) who received danazol treatment over a period of 1 to 6 years, weight gain, myalgia, headache, abnormal liver function tests, anxiety, and microscopic hematuria were reported.^42^ While weight gain ranged from 2.3 kg to 4.5 kg on average, it was reported to be as high as 18.1 kg in occasional patients. The authors concluded that patients treated with higher doses of danazol experienced AEs more frequently. Although AE severity was not linked to dosage, it was directly related to treatment duration. As reported in 1 US-based study, HAE patients exposed to AAs had a 28% increase in non-HAE comorbidities, including anxiety/depression, muscle cramps, obesity, and hyperlipidemia, compared to matched controls (p = 0.04).^83^ Additionally, each gram increase in AA exposure per month was associated with a 12% increase in comorbidities (p < 0.01).

Three studies and 15 case reports/series reported on patients who had received LTP with AA for at least 5 years (Fig. 3). Reported long-term consequences included virilization, such as menstrual irregularities and hirsutism, liver damage (including liver adenoma, hepatic lesions, and steatosis), and CV morbidity. Hepatocellular carcinoma, the most common primary liver cancer in the United States (standardized incidence of 5.49 per 100,000 people from 2001 to 2021),^103^ is a rare but serious adverse effect associated with LTP for HAE.^68^ Case reports highlighted 4 patients who developed hepatocellular carcinoma after receiving danazol for over 10 years at daily doses ranging from 200 mg to 400 mg.^104^, ^105^, ^106^, ^107^ Importantly, these patients had no other risk factors for hepatocellular carcinoma (eg, cirrhosis) and generally exhibited normal liver function, with only minor abnormalities at time of diagnosis. In addition, the occurrence of hepatocellular adenomas in patients receiving LTP with danazol was also reported.^37^^,^^38^ In 1 case study, 3 of 11 patients who had received LTP with danazol for over 10 years developed hepatocellular adenomas.^38^ The authors suggested that LTP with danazol may be a greater risk than previously recognized, even at low doses. In a follow-up report, a fourth patient who had developed hepatocellular adenoma after 17 years of danazol use was described.^37^ Notably, in 2 of the cases, the adenomas regressed following the discontinuation of danazol, providing further evidence of a potentially causative role.^37^^,^^38^

**Fig. 3:**
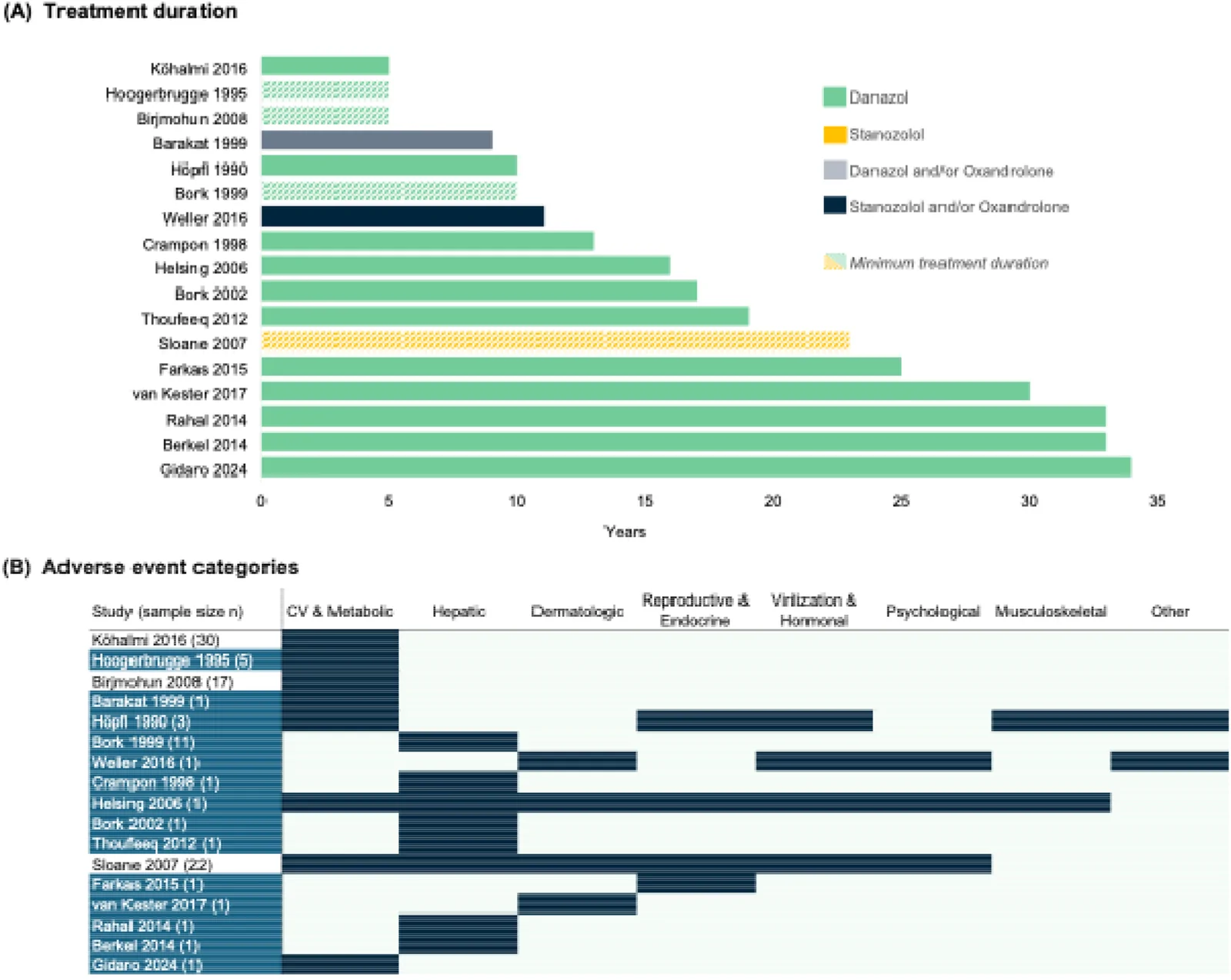
**Adverse events with ≥5 years of attenuated androgen use**. Key: CV, cardiovascular; PCOS, polycystic ovary syndrome. Note: Diagonal stripes indicate minimum treatment duration. Adverse events were categorized in the following way – CV & metabolic (hypertension, weight gain, abnormal serum lipid profile, hypercholesterolemia, increased coagulation activation, myocardial hypertrophy, erythrocytosis, elevated cholesterol), hepatic (fatty liver, steatosis, transaminase elevations, hepatocellular adenoma, hepatocellular carcinoma, hepatocellular focal nodular hyperplasia, liver damage), dermatologic (acne, urticaria, erythema marginatum), reproductive & endocrine (menstrual irregularities, atrophic uterus, miscarriages, post-menopausal bleeding, PCOS), virilization & hormonal (voice changes, deepening of voice, virilization, hirsutism), psychological (mood changes, psychological morbidity, emotional distress, fatigue), musculoskeletal (myalgia, muscle cramps), other (diffuse abdominal symptoms, increased sweating). Studies in blue represent case reports/series and studies in white represent other study types, including 2 observational studies (Köhalmi 2016, Sloane 2007) and 1 clinical trial (Birjmohun 2008).

### Adverse effects in women

A primary concern in women treated with AA LTP is their virilizing effect and their impact on menstrual cycles.^20^ While many studies did not report AEs under the broader term “virilization”, specific virilization effects were often noted. Where virilization was reported, the definition of what AEs were considered virilizing effects was rarely stated. In addition, some studies reported virilizing effects across the whole study population (including male patients), without stratifying by sex.

Three observational studies specifically analysed AEs in female HAE patients.^20^^,^^82^^,^^89^ While Steiner et al (2016) conducted a stratified analysis of female HAE patients receiving LTP with danazol, their focus was on co-morbidities (eg, CV and psychological morbidity) rather than virilization outcomes.^82^ In addition, 5 clinical trials, 16 observational studies, and 16 case reports/series captured the prevalence for AEs commonly included under the broader term of virilization (Table 5). It should be noted that the observed variation across studies may be the result of an inconsistent application of the definition of virilization effects.

The prevalence of virilization across identified studies ranged from 0% to 84%.^20^^,^^46^^,^^54^ Virilization rates may be under-reported in studies that do not specifically report virilization as a defined outcome. For example, Cicardi et al (1991) reported no virilization effects, but separately reported that 80% of pre-menopausal women with a uterus experienced menstrual irregularities and one woman experienced breast changes, symptoms often included under the umbrella term of virilization.^54^ Across included studies, generally 24%–100% of women experienced menstrual irregularities. Virilization and menstrual irregularities appear to be likely dose-dependent^50^^,^^53^^,^^69^ and may resolve with dose reduction.^28^^,^^29^^,^^54^^,^^89^^,^^90^ However, certain AEs may persist even at the minimal effective dose. One study reported that female patients receiving a mean daily danazol dose of 131.7 mg [range: 33 mg–200 mg] often experienced AEs such as hirsutism, weight gain, and menstrual disturbances. The menstrual disturbances were resolved after AAs were discontinued.^20^

### CV risk factors and events

CV risk factors and events were reported across 13 studies, including one clinical trial and 12 observational studies (Table 6; Table 7). In studies comparing patients with HAE receiving LTP with AAs with those not receiving AAs, low-density lipoprotein cholesterol (LDL-C) levels were found to be significantly higher (p < 0.05) and high-density lipoprotein cholesterol (HDL-C) to be significantly lower (p < 0.001) among patients receiving LTP with AAs.^30^, ^31^, ^32^ Similarly, HAE patients treated with AAs showed a trend towards higher total cholesterol levels, though the difference did not reach statistical significance.^30^, ^31^, ^32^^,^^53^ A retrospective cross-sectional study of 650 patients with HAE found that elevated cholesterol levels were correlated with the duration of AA exposure (p < 0.0001), but not with AA dose.^88^

Despite the increased prevalence of CV risk factors in HAE patients treated with AAs, available evidence was limited and did not show a clear association between AA exposure and an increase in adverse CV events. While comparative studies^32^^,^^87^ generally did not show an association between HAE LTP with AAs and an increased risk of CV events, several other reports linked CV events to LTP with AAs.^50^^,^^70^ For instance, a 34-year-old patient experienced a myocardial infarction after 16 years of taking danazol at an average daily dose of 200 mg, with a second myocardial infarction occurring 18 months later.^50^ In the same study, another patient without a prior diagnosis of hypertension experienced a stroke after 3 years on a daily danazol regimen of 400 mg.

### Patient and physician perspectives

Patient and physician attitudes and perceptions regarding the use of AAs were reported across 28 studies (including 1 clinical trial, 24 observational studies, and 3 case reports/series). The weighing of treatment options, by both patients and physicians, was heavily influenced by safety considerations (including both fear or experience of side effects),^20^^,^^33^^,^^34^^,^^49^^,^^50^^,^^51^^,^^57^^,^^59^^,^^65^^,^^74^^,^^81^, ^67^, ^71^, ^86^^,^^88^^,^^108^^,^^109^ cost barriers,^51^^,^^59^^,^^65^^,^^78^, ^79^, ^80^ and convenience.^59^^,^^74^^,^^78^, ^79^, ^80^, ^81^

Physicians’ personal experience and patient preferences were also noted to play a role when considering treatment options.^28^^,^^59^^,^^65^^,^^67^^,^^71^^,^^78^, ^79^, ^80^, ^81^ Patients and physicians cited androgen-related AEs and patient preferences as key reasons for switching to newer treatments.^71^

Physicians also noted that LTP with AAs is not typically initiated anymore in newly diagnosed patients with HAE, and individuals currently taking AAs have likely been on this treatment for decades.^71^ In 3 serial surveys of US-based physicians conducted by Riedl and colleagues, the reluctance to prescribe AAs for LTP has significantly increased over time, with unwillingness to prescribe rising from 18% in 2010 to 60% in 2019.^80^ Significant changes (p < 0.001) were seen in the reported prescribing of danazol (decreased from 55.8% in 2010 to 23.4% in 2013, and to 6.4% in 2019). In a recent United Kingdom (UK) consensus statement, physicians affirmed the role of AAs as a suitable LTP for some patients, while also emphasizing its unsuitability for specific patients such as children and pregnant women.^86^

In patient surveys capturing preferences around prophylactic treatment options for HAE, AAs had the most perceived AEs, the lowest median confidence, and the least overall positive impression of the medication, despite their oral route of administration.^49^^,^^59^^,^^67^^,^^73^^,^^77^, ^78^, ^79^, ^80^ In 1 study among patients with prior or current use of AAs, approximately 78% felt that AEs outweighed the benefits of treatment.^88^

## Discussion

This study provides a comprehensive synthesis of the current evidence on adverse health outcomes associated with AA LTP in patients with HAE. The large body of evidence we identified enabled us to further synthesize the available evidence with a particular focus on the significant impact on the female patients and the long-term risks associated with the use of AAs as a prophylaxis for HAE. Moreover, by integrating patient and physician perspectives, this review highlights the nuanced nature of LTP for HAE, providing a multidimensional understanding of AA use in current HAE management. The findings of this review underscore long-standing safety concerns about AAs and align with the shifting clinical landscape that favors newer, more targeted therapies.

The far-ranging impact of AA LTP on patients with HAE and associated adverse health outcomes were frequently highlighted in the identified literature. Across 76 studies reporting on AEs, AAs were consistently associated with a broad spectrum of unfavorable health outcomes. Increased body weight, menstrual irregularities, acne, liver damage, hirsutism, psychological morbidity, CV morbidity, and hypertension were among the most frequently reported AEs associated with AA use.

Where androgen type was reported, most evidence concerned danazol and, to a lesser extent, stanozolol; data on oxandrolone and methyltestosterone were sparse. Findings therefore predominantly reflect the androgenic safety profile of danazol and may not be reflective of the relatively milder androgenic safety profile of other agents. These findings are consistent with earlier literature reviews, which similarly identified significant toxicity concerns despite prophylactic efficacy.^4^

The dose- and duration-dependent nature of many adverse health outcomes was highlighted in numerous studies.^42^^,^^83^^,^^88^ While certain AEs (eg, weight gain or acne) appeared within just a few months of treatment initiation, others (eg, hepatic damage and changes in lipid profiles) were more likely with prolonged AA use. Importantly, the available evidence showed that liver toxicities, including hepatocellular carcinoma and hepatic adenoma, occurred even at the lowest effective dose.^38^^,^^37^^,^^104^, ^105^, ^106^, ^107^ These cases underscore the rare but serious risk of hepatocellular carcinoma associated with prolonged use of danazol as prophylaxis and puts into question the traditional dose safety thresholds for AAs. Hepatocellular carcinoma, a rare yet serious adverse health outcome, was detailed in 4 case studies, all involving LTP with danazol for over 10 years.^104^, ^105^, ^106^, ^107^ As these patients lacked an alternative etiology (eg, cirrhosis), a causative role of long-term exposure to danazol cannot be ruled out. These cases highlight the hepatotoxic risks associated with LTP using danazol and highlight the importance of regular liver monitoring. Collectively, the findings caution against extended high-dose exposure to AAs in HAE management and suggest that time-limited treatment may be warranted if AAs are used.

Female patients with HAE in particular face a disproportionate burden due to the virilizing effects of AAs. While AEs under the broader term “virilization” were infrequently reported, specific virilization effects were often noted. Where virilization was reported as an outcome, consistent definitions were often lacking. In addition, some studies reported virilizing effects across the study population in entirety without stratifying by sex. This explains the highly variable prevalence of virilization across studies, ranging from 0% to 84%, with menstrual irregularities occurring in up to 100% of women in some studies.^20^^,^^46^^,^^54^ Virilization rates may not be accurately represented in the available evidence. The sensitive nature of the topic, particularly for symptoms such as hirsutism or voice deepening, may contribute to women under-reporting these symptoms to the treating physician or study investigator due to fear of stigma. The lack of a standardized definition of virilization across studies limits the ability to assess the full impact on women and highlights a critical evidence gap. This underreporting may also obscure potential implications for fertility and fetal development, which, while beyond the scope of this review, were rarely noted or addressed in the reviewed studies. Lastly, although beyond the scope of this SLR, evidence suggests that long-term AA use may impact fertility and cause fetal virilization, as evidenced by reports of miscarriages.^92^

The totality of the identified literature confers strong evidence for alteration of CV risk factors such as elevated LDL-C and total cholesterol, as well as decreased HDL-C with AA LTP for HAE. However, evidence directly linking AA use to CV events remains limited and inconsistent.^32^^,^^50^^,^^53^^,^^70^^,^^83^ The sparsity of data is not unexpected, given the rarity of HAE, small sample sizes, and lack of long-term follow-up. While several retrospective studies did not report increased CV event rates, despite identifying unfavorable CV risk profiles, these findings should be interpreted in light of the methodological challenges inherent to rare outcomes in a rare disease patient population. There remains a gap in studies that characterize long-term CV risks associated with AA use, including a lack of comparative studies, larger cohort studies, and studies with standardized endpoints.

Patient and physician perspectives consistently highlighted concerns around the tolerability and safety of AAs. In a series of 3 consecutive US-based surveys, the reluctance to prescribe AAs for LTP has significantly increased from 18% in 2010 to 60% in 2019.^80^ US-based physicians also reported a sharp decline in issuing danazol prescriptions – from 55.8% in 2010 to just 6.4% in 2019 – mirroring the increasing availability of modern therapies.^78^, ^79^, ^80^ Nearly 80% of patients surveyed felt that the risks of AAs outweighed the benefits.^88^ Despite these concerns, AAs remain in use, particularly in settings with limited access to newer, targeted treatments or for HAE patients on LTP with AAs with long-standing stable disease. These findings emphasize the importance of integrating real-world perspectives and shared decision-making into clinical guidelines.

The findings of this study align with current guidelines, which position AAs as a non-preferred, second-line option for HAE LTP.^10^ While AAs have some efficacy in HAE attack prevention, their use is limited due to their side effect profiles and the availability of targeted, more effective LTP options. Even in patients where AA use is not contraindicated, their unfavorable safety profiles and the elevated likelihood of irreversible long-term risks such as CV morbidity and hepatocellular carcinoma are contrary to the WAO/EAACI goal of treatment with the goal of normalizing HAE patients’ lives. Therefore, clinical use of AAs must be accompanied by close monitoring and a proactive strategy to transition patients to safer alternatives when feasible.

## Limitations

The findings of our study should be interpreted in light of several limitations. Few prospective controlled trials for the use of AAs as LTP for HAE were identified, which limited our ability to establish causality between AA use and reported adverse health outcomes. The majority of included studies were observational or descriptive. The included studies varied significantly in terms of patient demographics (eg, sex) and study design, including AA dosing regimens, treatment duration, and follow-up periods. This heterogeneity precludes pooling study data, thus allowing only a qualitative synthesis of results. This review did not include a formal risk of bias assessment, which limits the ability to weight findings according to methodological rigor and study quality. Further, due to potential publication bias across all included studies, findings may not reflect the true relative prevalence of adverse health outcomes. Severe AEs (eg, liver damage) may be over-represented, while less severe AEs (eg, acne and menstrual irregularities) may be under-reported. Many studies lacked sex-specific reporting, particularly virilization effects, which limits insight into the true treatment burden of LTP with AAs in female patients with HAE. Lastly, the rarity of HAE and the infrequent use of AAs in current clinical practice limit the sample sizes and generalizability of included studies.

## Conclusions

This study identified a large but heterogeneous body of evidence documenting adverse health outcomes and long-term consequences of prophylaxis with AAs in patients with HAE. Findings showed that, while AAs have been used for LTP in HAE, they are linked to a multitude of adverse health outcomes, including weight gain, virilization, liver toxicity, and CV risks. Despite increased reluctance of physicians to use AAs in clinical practice and newer prophylactic agents now being available, AAs are still being used due to various reasons, most notably access issues and familiarity with the treatment. Improved access to targeted therapies and more research into the long-term CV risks of AAs are paramount to ensure accurate clinical decision-making and optimize patient care.

## Ethics declaration

Not applicable.

## Author contributions

All authors have made substantial contributions to conception and design, or acquisition of data, or analysis and interpretation of data. All authors were involved in drafting the manuscript or revising it critically for important intellectual content. All authors have given final approval of the version to be published.

## Submission declaration

This study was funded by BioCryst Pharmaceuticals, Inc. The work has not been previously published and is not being considered for publication by another journal. An earlier version of this study was presented in poster form at EAACI 2024 (#D1.458). All authors, along with the relevant authorities at the institution where the work was conducted, have approved the submission.

## Disclosure of generative AI and AI-assisted technologies

ChatGPT (OpenAI) was only used for language refinement and grammar checking. The authors reviewed, edited, and take full responsibility for the final content of the manuscript.

## Role of funding source

BioCryst provided financial support for the conduct of this study and was involved in the study design; interpretation of data; in the writing of the report; and in the decision to submit the article for publication.

## Potential competing interests

Dr Markus Magerl has received funding or compensation in the form of grants or contracts, consulting fees, honoraria, and/or travel support from Astria, Argo, BioCryst, CSL Behring, Intellia, Kalvista, Octopharma, Otsuka, Pharvaris, and Takeda; served on data safety monitoring or advisory boards for BioCryst, CSL Behring, KalVista, Pharvaris, Octopharma, and Takeda; and holds leadership or fiduciary roles in steering committees for BioCryst, CSL Behring, and Otsuka. Dr Anete S Grumach has received funding or compensation in the form of grants or contracts, consulting fees, honoraria, and/or travel support from Astra Zeneca, BioCryst, the Brazilian Entity of Research (CNPq), CSL Behring, Kalvista, Multicare, Pharvaris, Pint-Pharma, and Takeda; served on data safety monitoring or advisory boards for CSL Behring, Takeda, Pharvaris, and Pint-Pharma; and hold leadership or fiduciary roles in the ACARE steering committee, Brazilian group of Primary Immunodeficiency (BRAGID), Group of Study on Hereditary Angioedema (GEBRAEH), and the HAE Dept of Brazilian Association of Allergy and Immunology (ASBAI). Dr Marcin Stobiecki has received funding or compensation in the form of consulting fees and/or honoraria from BioCryst, CSL Behring, Genesis Pharma, KalVista, Pharming, Pharvaris, Takeda and Swixx. Dr Sorena Kiani-Alikhan has received funding or compensation in the form of consulting fees, honoraria, and/or travel support from BioCryst, Pharvaris, Otsuka, and Takeda. Adam Gordois, Dr Maria Pedrosa, Dr Sandra Nestler-Parr, and Dr Patrick Gillard receive compensation and stock from BioCryst Pharmaceutical as full time employees. Noemi Fluetsch Brennan, Nikolina Boskovic, and Dr Lisa Bloudek are employed by Curta, an organization that received funding from BioCryst related to this study. Dr Lisa Bloudek is an equity shareholder in Curta, Inc.
