# Supplementary material for: Adverse health outcomes and patient and physician perspectives of attenuated androgen use in hereditary angioedema
Source: World Allergy Organ J. 2026 Sep 22;19(10):101471. doi: 10.1016/j.waojou.2026.101471 (PMC13675641; doi:10.1016/j.waojou.2026.101471)
Supplement: Supplementary file 1 [file mmc1.doc]

**SUPPLEMENT**

**Supplemental Table A. MEDLINE search strategy**

| **Search** | **Terms** |
| --- | --- |
| #1: Attenuated Androgens | "danazol"[MeSH Terms] OR danocrine[Text Word] OR "stanozolol"[MeSH Terms] OR "winstrol"[Text Word] OR "oxandrolone"[MeSH Terms] OR oxandrolone[Text Word] OR "oxandrin"[Text Word] OR "Androgens/chemical synthesis"[MeSH] OR "Androgens"[nm] OR "Androgens/therapeutic use"[MAJR] OR "Androgens"[Mesh] OR “androgen*”[tiab] OR "Danazol/therapeutic use"[MeSH] OR "Stanozolol/therapeutic use"[MeSH] OR " oxandrolone/therapeutic use"[MeSH] OR " Methyltestosterone/therapeutic use"[MeSH] OR “Methyltestosterone”[MeSH] OR “Methyltestosterone”[tiab] |
| #2: HAE | "Angioedemas, Hereditary/therapy"[MAJR] OR "Angioedemas, Hereditary"[Mesh] OR "Angioedemas, Hereditary/drug therapy"[MAJR] OR "Angioedemas, Hereditary/prevention and control"[MAJR] OR "hereditary angioedema"[tiab:~0] OR "hereditary angio-oedema"[tiab:~0] OR "HAE"[tiab] |
| #3: Attenuated Androgen Use In HAE | #1 AND #2 |
| #4: Exclusions | #3 NOT (anabolic[tiab] OR anti-androgen*[tiab] OR "prostate cancer"[tiab:~0] OR Klinefelter[tiab] OR PCOS[tiab] OR "polycystic ovary"[tiab:~0] OR alopecia[tiab] OR chorioretinopathy[tiab] OR turtle*[tiab] OR rats[tiab] OR rat[tiab] OR macaques*[tiab] OR mice[tiab] OR mouse[tiab] OR rabbit*[tiab]) |
| #5: Study/ Publication Type Exclusions | #4 NOT ("comment"[pt] OR "editorial"[pt] OR "clinical trial protocol"[pt]) |
| #6: Non-Human Exclusions | #5 NOT ("Animals"[Mesh] NOT "Humans"[Mesh]) |

**Supplemental Table B. EMBASE search strategy**

| **Search** | **Terms** |
| --- | --- |
| #1: Attenuated androgens | 'danazol'/exp OR 'danazol':ti,ab OR 'danocrine':ti,ab OR 'stanozolol'/exp OR 'winstrol':ti,ab OR 'oxandrolone'/exp OR 'oxandrolone':ti,ab OR 'oxandrine':ti,ab OR 'methyltestosterone'/exp OR 'methyltestosterone':ti,ab OR 'androgen'/exp OR 'androgen':ti,ab |
| #2: Attenuated Androgen Use In HAE | 'angioneurotic edema' OR 'hereditary angioedema':ti,ab OR "hereditary angio-oedema":ti,ab OR 'hae':ti,ab |
| #3: Attenuated Androgen Use in HAE and Other Conditions | #1 AND #2 |
| #4: Exclusions | #3 NOT ('anabolic':ti,ab OR 'anti-androgen*':ti,ab OR 'prostate cancer':ti,ab OR 'klinefelter':ti,ab OR pcos:ti,ab OR 'polycystic ovary':ti,ab OR 'alopecia':ti,ab OR 'chorioretinopathy':ti,ab OR 'turtle*':ti,ab OR 'rats':ti,ab OR rat:ti,ab OR 'macaques*':ti,ab OR 'mice':ti,ab OR 'mouse':ti,ab OR 'rabbit*':ti,ab) |
| #5: Study/ Publication Type Exclusions | #4 NOT ([editorial]/lim OR [letter]/lim OR [note]/lim OR [short survey]/lim OR [conference abstract]/lim) |
| #6: Non-Human Exclusions | #5 NOT (('animal'/exp OR 'nonhuman'/exp OR 'animal experiment'/exp OR 'animal model'/exp OR 'in vitro study'/de) NOT 'human'/exp) |

**Supplemental Table C.** PRISMA checklist

| **Section and Topic** | **Item #** | **Checklist item** | **Location where item is reported** |
| --- | --- | --- | --- |
| **TITLE** | | |  |
| Title | 1 | Identify the report as a systematic review. |  |
| **ABSTRACT** | | |  |
| Abstract | 2 | See the PRISMA 2020 for Abstracts checklist. |  |
| **INTRODUCTION** | | |  |
| Rationale | 3 | Describe the rationale for the review in the context of existing knowledge. |  |
| Objectives | 4 | Provide an explicit statement of the objective(s) or question(s) the review addresses. |  |
| **METHODS** | | |  |
| Eligibility criteria | 5 | Specify the inclusion and exclusion criteria for the review and how studies were grouped for the syntheses. |  |
| Information sources | 6 | Specify all databases, registers, websites, organisations, reference lists and other sources searched or consulted to identify studies. Specify the date when each source was last searched or consulted. |  |
| Search strategy | 7 | Present the full search strategies for all databases, registers and websites, including any filters and limits used. |  |
| Selection process | 8 | Specify the methods used to decide whether a study met the inclusion criteria of the review, including how many reviewers screened each record and each report retrieved, whether they worked independently, and if applicable, details of automation tools used in the process. |  |
| Data collection process | 9 | Specify the methods used to collect data from reports, including how many reviewers collected data from each report, whether they worked independently, any processes for obtaining or confirming data from study investigators, and if applicable, details of automation tools used in the process. |  |
| Data items | 10a | List and define all outcomes for which data were sought. Specify whether all results that were compatible with each outcome domain in each study were sought (e.g. for all measures, time points, analyses), and if not, the methods used to decide which results to collect. |  |
|  | 10b | List and define all other variables for which data were sought (e.g. participant and intervention characteristics, funding sources). Describe any assumptions made about any missing or unclear information. |  |
| Study risk of bias assessment | 11 | Specify the methods used to assess risk of bias in the included studies, including details of the tool(s) used, how many reviewers assessed each study and whether they worked independently, and if applicable, details of automation tools used in the process. |  |
| Effect measures | 12 | Specify for each outcome the effect measure(s) (e.g. risk ratio, mean difference) used in the synthesis or presentation of results. |  |
| Synthesis methods | 13a | Describe the processes used to decide which studies were eligible for each synthesis (e.g. tabulating the study intervention characteristics and comparing against the planned groups for each synthesis (item #5)). |  |
|  | 13b | Describe any methods required to prepare the data for presentation or synthesis, such as handling of missing summary statistics, or data conversions. |  |
|  | 13c | Describe any methods used to tabulate or visually display results of individual studies and syntheses. |  |
|  | 13d | Describe any methods used to synthesize results and provide a rationale for the choice(s). If meta-analysis was performed, describe the model(s), method(s) to identify the presence and extent of statistical heterogeneity, and software package(s) used. |  |
|  | 13e | Describe any methods used to explore possible causes of heterogeneity among study results (e.g. subgroup analysis, meta-regression). |  |
|  | 13f | Describe any sensitivity analyses conducted to assess robustness of the synthesized results. |  |
| Reporting bias assessment | 14 | Describe any methods used to assess risk of bias due to missing results in a synthesis (arising from reporting biases). |  |
| Certainty assessment | 15 | Describe any methods used to assess certainty (or confidence) in the body of evidence for an outcome. |  |
| **RESULTS** | | |  |
| Study selection | 16a | Describe the results of the search and selection process, from the number of records identified in the search to the number of studies included in the review, ideally using a flow diagram. |  |
|  | 16b | Cite studies that might appear to meet the inclusion criteria, but which were excluded, and explain why they were excluded. |  |
| Study characteristics | 17 | Cite each included study and present its characteristics. |  |
| Risk of bias in studies | 18 | Present assessments of risk of bias for each included study. |  |
| Results of individual studies | 19 | For all outcomes, present, for each study: (a) summary statistics for each group (where appropriate) and (b) an effect estimate and its precision (e.g. confidence/credible interval), ideally using structured tables or plots. |  |
| Results of syntheses | 20a | For each synthesis, briefly summarise the characteristics and risk of bias among contributing studies. |  |
|  | 20b | Present results of all statistical syntheses conducted. If meta-analysis was done, present for each the summary estimate and its precision (e.g. confidence/credible interval) and measures of statistical heterogeneity. If comparing groups, describe the direction of the effect. |  |
|  | 20c | Present results of all investigations of possible causes of heterogeneity among study results. |  |
|  | 20d | Present results of all sensitivity analyses conducted to assess the robustness of the synthesized results. |  |
| Reporting biases | 21 | Present assessments of risk of bias due to missing results (arising from reporting biases) for each synthesis assessed. |  |
| Certainty of evidence | 22 | Present assessments of certainty (or confidence) in the body of evidence for each outcome assessed. |  |
| **DISCUSSION** | | |  |
| Discussion | 23a | Provide a general interpretation of the results in the context of other evidence. |  |
|  | 23b | Discuss any limitations of the evidence included in the review. |  |
|  | 23c | Discuss any limitations of the review processes used. |  |
|  | 23d | Discuss implications of the results for practice, policy, and future research. |  |
| **OTHER INFORMATION** | | |  |
| Registration and protocol | 24a | Provide registration information for the review, including register name and registration number, or state that the review was not registered. |  |
|  | 24b | Indicate where the review protocol can be accessed, or state that a protocol was not prepared. |  |
|  | 24c | Describe and explain any amendments to information provided at registration or in the protocol. |  |
| Support | 25 | Describe sources of financial or non-financial support for the review, and the role of the funders or sponsors in the review. |  |
| Competing interests | 26 | Declare any competing interests of review authors. |  |
| Availability of data, code and other materials | 27 | Report which of the following are publicly available and where they can be found: template data collection forms; data extracted from included studies; data used for all analyses; analytic code; any other materials used in the review. |  |

**Supplemental Table D. Included case reports/series**

| **Study** | **Study design** | **Geography** | **N** | **Intervention** | **Age, years** | **Female, %** | **Duration of AA use** |
| --- | --- | --- | --- | --- | --- | --- | --- |
| Aguilar 2000^66^ | Case report | US | 1 | Stanozolol / Danazol | 16 | 100 | Current treatment course: 1.5 years |
| Akoglu 2017^110^ | Case series | Turkey | 3 | Danazol | Range: [31, 58] | 66.6 | 2 - 5 years |
| Altman 2006^63^ | Case report | Canada | 1 | Danazol | 22 | 100 | 6 weeks |
| Azzam 2015^111^ | Case series | Lebanon | 1 | Danazol | 25 | 0 | – |
| Barakat 1999^35^ ^a^ | Case report | US | 1 | Danazol / Oxandrolone | 14 | 100 | 9 years |
| Barakat 1993^34^ ^a^ | Case report | US | 1 | Danazol | 5 | 100 | 2 years |
| Berkel 2014^56^ | Case report | The Netherlands | 1 | Danazol | 75 | 100 | 33 years |
| Bork 2002^60^ ^b^ | Case report | Germany | 1 | Danazol | 45 | 0 | 17 years |
| Bork 1999^37^ ^b^ | Case series | Germany | 41 | Danazol | – | – | >10 years (n=11) |
| Bretza 1980^112^ | Case report | US | 1 | Danazol | 28 | 0 | – |
| Bui Van 2012^113^ | Case report | Vietnam | 1 | Danazol | 40 | 0 | 3 months |
| Chen 2014^114^ | Case report | China | 1 | Danazol | 50 | 100 | 8 months |
| Crampon 1998^57^ | Case report | The Netherlands | 1 | Danazol | 34 | 100 | 13 years |
| Dertlioglu 2013^115^ | Case series | Turkey | 2 | Danazol | Range: [16, 28] | 100 | 4 months |
| Duhra 1990^100^ | Case report | United Kingdom | 1 | Danazol | 38 | 100 | 2 years |
| Farkas 2015^82^ | Case report | Hungary | 1 | Danazol | 34 | 100 | 25 years |
| Gidaro 2024^116^ | Case report | Italy | 1 | Stanozolol / Danazol | 57 | 0 | 34 years |
| Gower 2012^101^ | Case report | US | 3 | Danazol: 66.6% Unknown: 33.3% | Median [Range]: 28 [24, 41] | 100 | 1 year |
| Hammami 2011^117^ | Case report | Tunisia | 1 | Danazol | 14 | 0 | 1 |
| Helsing 2006^102^ | Case report | Norway | 1 | Danazol | 32 | 100 | 16 years |
| Herrmann 2004^103^ | Case report | United Kingdom | 1 | Danazol | 31 | 100 | 2 years |
| Heusse 2008^118^ | Case report | US | 1 | Danazol | 31 | – | – |
| Hintner 1979^38 c^ | Case series | Germany | 4 | Danazol | Range: [9, 58] | 1 | 8 - 34 weeks |
| Hoarau 2024^72^ | Case series | France | 2 | Danazol | Range: [21, 52] | 50 | ≥ 20 years |
| Hoogerbrugge 1995^119^ | Case series | The Netherlands | 5 | Danazol | – | 80 | >5 years |
| Höpfl 1990^104^ | Case series | Austria | 3 | Danazol | Median [Range]: 35 [19, 69] | 33.3 | 10 years |
| Hory 1985^120^ | Case report | France | 1 | Danazol | 18 | 0 | 0.3 years |
| Jindal 2024^105^ | Case report | India | 1 | Stanozolol and tranexamic acid | 17 | 1 | – |
| Lei 2011^106^ | Case report | Taiwan | 2 | Danazol | – | 50 | – |
| Marrawani 2024^99^ | Case report | Palestine | 1 | Danazol | 24 | 100 | – |
| Maurer 2022^24^ | Case series | Germany | 10 | Danazol: 80.0%  Oxandrolone: 20.0% | Median [Range]: 51 [31, 76] | 30.0 | Median [Range]: 16.5 [1.5, 36] |
| Pastó Cardona 2004^107^ | Case series | Spain | 8 | Danazol | 48.4 [Range: 25, 76] | 62.5 | – |
| Rahal 2014^58^ | Case report | France | 1 | Danazol | 57 | 0 | 33 years |
| Rajagopal 1981^121^ | Case report | United Kingdom | 1 | Danazol | 10 | 0 | 10 months |
| Spaulding 1979^122^ | Case series | Canada | 1 | Danazol | 40 | 100 | Approximately 1 year |
| Suh 1995^108^ | Case series | Korea | 1 | Danazol | 20 | 100 | 8 weeks |
| Tappeiner 1979^39 c^ | Case report | Austria | 1 | Danazol | 8 | 0 | 11 months |
| Thoufeeq 2012^59^ | Case report | United Kingdom | 1 | Danazol | 75 | 0 | 19 years |
| van Kester 2017^123^ | Case report | The Netherlands | 1 | Danazol | 55 | 100 | 30 years |
| Weller 2016^109^ | Case report | Germany | 1 | Oxandrolone /  Stanozolol | 25 | 100 | 11 years |

Key: AA, attenuated androgen; N, total number; US, United States of America

^a^ Barakat 1999 reports on the same case described in Barakat 1993 with additional follow-up.

^b^ Bork 2002 provides updates to cases described in Bork 1999 and reports on one additional case.

^c^ Hintner 1979 includes a case previously described in Tappeiner 1979.

**Supplemental Reference List**

110.	Akoglu G, Kesim B, Yildiz G, Metin A. Outcomes of long term treatments of type I hereditary angioedema in a Turkish family. *An Bras Dermatol*. 2017;92(5):655-660. doi:10.1590/abd1806-4841.20175899

111.	Azzam R, Boutros J, Irani C. Hereditary angioedema: a literature review and national management guidelines. *J Med Liban*. 2015;63(2):97-101. doi:10.12816/0012558

112.	Bretza JA, Novey HS, Vaziri ND, Warner AS. Hypertension: a complication of danazol therapy. *Arch Intern Med*. 1980;140(10):1379-80. doi:10.1001/archinte.140.10.1379

113.	Bui Van K, Nguyen Van D. The first probable case of hereditary angioedema in Vietnam. *Allergy Asthma Immunol Res*. 2012;4(3):165-7. doi:10.4168/aair.2012.4.3.165

114.	Chen X, Yang YX, Liu YL, Gan HT, Wen ZH. Hereditary angioedema: a rare cause of recurrent abdominal pain. *Pak J Med Sci*. 2014;30(5):1147-9. doi:10.12669/pjms.305.5524

115.	Dertlioglu SB, Cicek D, Yesilova Y. A rare cause of recurrent abdominal pain: three familial cases with hereditary angioedema. *Indian J Dermatol Venereol Leprol*. 2013;79(1):105-7. doi:10.4103/0378-6323.104681

116.	Gidaro A, La Cava L, Donadoni M, et al. Lanadelumab in a kidney transplant patient with hereditary angioedema due to C1-inhibitor deficiency and high cardiovascular risk - a case report. *Front Immunol*. 2024;15:1472390. doi:10.3389/fimmu.2024.1472390

117.	Hammami S, Harrathi K, Hadded S, et al. Hereditary angio-oedema: long-term prophylaxis with danazol. *Respiratory Medicine CME*. 2011;4(1):15-16. doi:https://doi.org/10.1016/j.rmedc.2010.01.006

118.	Heusse JL, Claude O, Lantieri L. [Can one propose aesthetic surgery to one male or female patient with an hereditary angio-oedema?]. *Ann Chir Plast Esthet*. 2008;53(3):289-92. Peut-on proposer un acte de chirurgie esthétique à un(e) patient(e) atteint(e) d'un oedème angioneurotique? doi:10.1016/j.anplas.2007.05.011

119.	Hoogerbrugge N, Jansen H, Birkenhäger JC. Danazol in the treatment of endometriosis and hereditary angio-oedema. *J Intern Med*. 1995;237(6):603-4. doi:10.1111/j.1365-2796.1995.tb00892.x

120.	Hory B, Blanc D, Boillot A, Panouse-Perrin J. Guillain-Barré syndrome following danazol and corticosteroid therapy for hereditary angioedema. *Am J Med*. 1985;79(1):111-4. doi:10.1016/0002-9343(85)90553-4

121.	Rajagopal C, Harper JR. Successful use of danazol for hereditary angio-oedema. *Arch Dis Child*. 1981;56(3):229-30. doi:10.1136/adc.56.3.229

122.	Spaulding WB. Myalgia and elevated creatine phosphokinase with danazol in hereditary angioedema. *Ann Intern Med*. 1979;90(5):854. doi:10.7326/0003-4819-90-5-854_2

123.	van Kester MS, Bergman W, Lavrijsen AP. A female with hereditary angioedema developing wheals: a case report. *Acta Derm Venereol*. 2017;97(2):285-286. doi:10.2340/00015555-2490
